# Development and validation of machine learning models to predict survival of patients with resected stage-III NSCLC

**DOI:** 10.3389/fonc.2023.1092478

**Published:** 2023-03-13

**Authors:** Long Jin, Qifan Zhao, Shenbo Fu, Fei Cao, Bin Hou, Jia Ma

**Affiliations:** ^1^ Department of Radiation Oncology, Shaanxi Provincial People’s Hospital, Xi’an, China; ^2^ School of Material Science & Engineering, Huazhong University of Science and Technology, Wuhan, China; ^3^ Department of Radiation Oncology, Shaanxi Provincial Cancer Hospital, Xi’an, China; ^4^ Department of Oncology, Shaanxi Provincial People’s Hospital, Xi’an, China; ^5^ Department of Thoracic Surgery, Shaanxi Provincial People’s Hospital, Xi’an, China; ^6^ Shaanxi Provincial People’s Hospital, Xi’an, China

**Keywords:** non-small cell lung cancer (NSCLC), stage-III, machine learning, survival predication, treatment recommendation, adjuvant therapy

## Abstract

**Objective:**

To compare the performance of three machine learning algorithms with the tumor, node, and metastasis (TNM) staging system in survival prediction and validate the individual adjuvant treatment recommendations plan based on the optimal model.

**Methods:**

In this study, we trained three machine learning madel and validated 3 machine learning survival models-deep learning neural network, random forest and cox proportional hazard model- using the data of patients with stage-al3 NSCLC patients who received resection surgery from the National Cancer Institute Surveillance, Epidemiology, and End Results (SEER) database from 2012 to 2017,the performance of survival predication from all machine learning models were assessed using a concordance index (c-index) and the averaged c-index is utilized for cross-validation. The optimal model was externally validated in an independent cohort from Shaanxi Provincial People’s Hospital. Then we compare the performance of the optimal model and TNM staging system. Finally, we developed a Cloud-based recommendation system for adjuvant therapy to visualize survival curve of each treatment plan and deployed on the internet.

**Results:**

A total of 4617 patients were included in this study. The deep learning network performed more stably and accurately in predicting stage-iii NSCLC resected patients survival than the random survival forest and Cox proportional hazard model on the internal test dataset (C-index=0.834 vs. 0.678 vs. 0.640) and better than TNM staging system (C-index=0.820 vs. 0.650) in the external validation. The individual patient who follow the reference from recommendation system had superior survival compared to those who did not. The predicted 5-year-survival curve for each adjuvant treatment plan could be accessed in the recommender system *via* the browser.

**Conclusion:**

Deep learning model has several advantages over linear model and random forest model in prognostic predication and treatment recommendations. This novel analytical approach may provide accurate predication on individual survival and treatment recommendations for resected Stage-iii NSCLC patients.

## 1 Introduction

**Figure 1:**
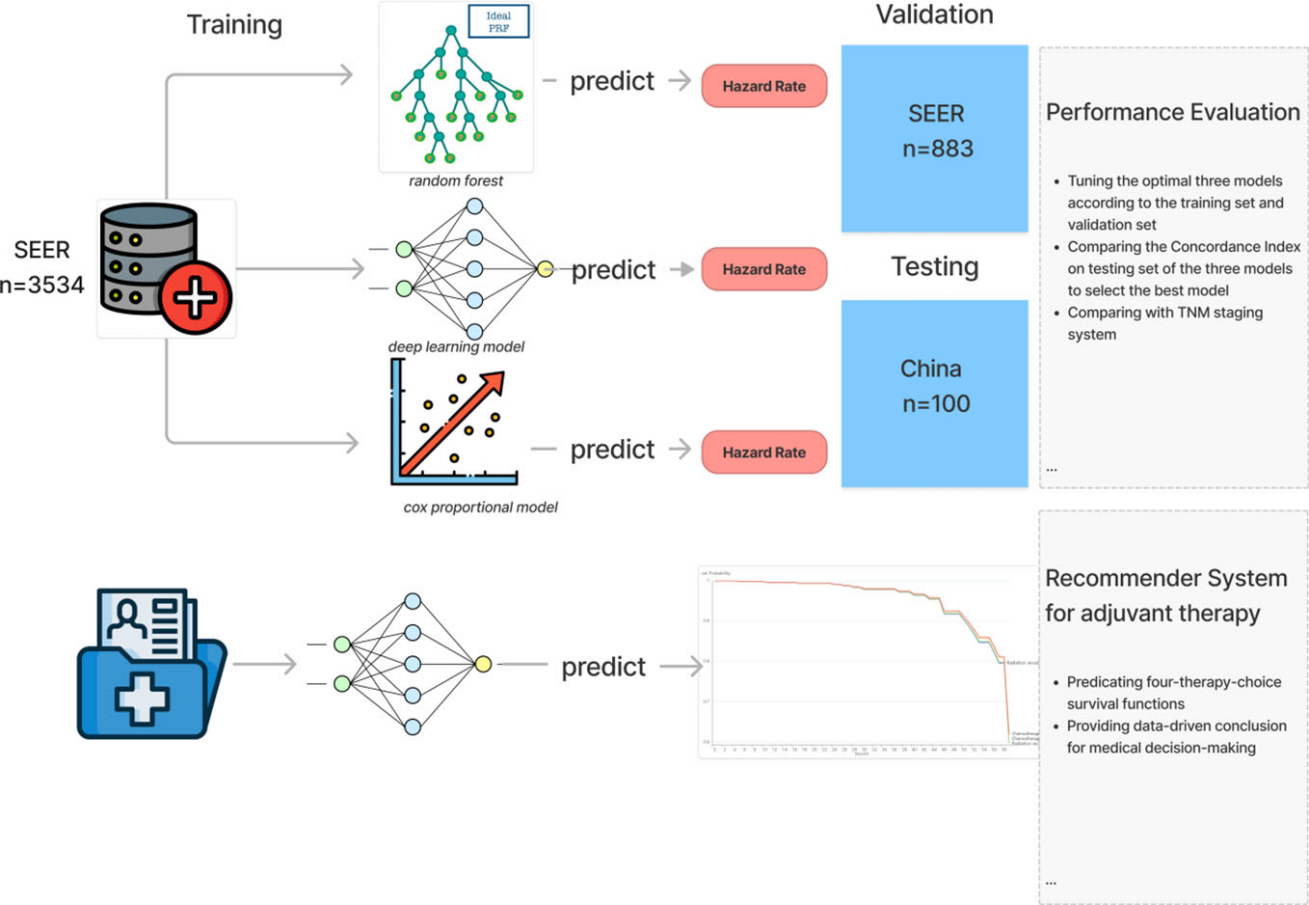
Diagram of the training and recommendation procedure.

Stage-iii non-small cell lung cancer (NSCLC) accounts for about 1/4 to 1/3 of total lung cancer and is a very heterogeneous disease with a discouraging clinical prognosis, the 5-year survival rate of NSCLC is only 15%-40% (1). For operable stage-iii lung cancer patients, surgery-based comprehensive treatment is recommended. However, even after radical tumor resection, there is still a high risk of recurrence and metastasis, so adjuvant therapy after surgery is required to improve long-term survival probability. Postoperative adjuvant therapy mainly includes adjuvant chemotherapy, radiotherapy and targeted therapy. Among them, adjuvant targeting is mainly aimed at the EGFR-amplified non-small cell lung cancer patients. Targeted therapy can improve its prognosis, but the proportion of this population is relatively low, only 9% of the total non-small cell lung cancer patients (2). For the vast majority of patients with EGFR-negative stage-iii lung cancer, studies have shown that postoperative chemotherapy (POCT) can improve the 5-year survival rate by 5% (3). Other researches confirm that the value of postoperative radiotherapy for high-risk subgroups (4–6), While the results of the meta-analysis in 1998 determines that postoperative adjuvant radiotherapy is not recommended for patients with stage I-IIIB (N0-N1) (7). In addition, the 2020 Lung ART study suggests that adjuvant radiotherapy is not recommended for patients with N2 after lung cancer surgery (8). Therefore, whether postoperative radiotherapy has a beneficial effect on overall survival (OS) is controversial. In the current clinical practice, the formulation and implementation of adjuvant chemotherapy and radiotherapy treatment plans are mainly based on the TNM staging system. Therefore, there are two drawbacks. The first defect is that only three clinical indicators of patients T, N, and M are considered to guide the clinical treatment of patients while ignoring other important characteristics of patients such as physiological characteristics (age, gender) and Other important clinical characteristics (surgical method, primary tumor location, tumor grade, number of positive lymph nodes (LNs), number of LNs examined, and adjuvant therapy methods). Secondly, the TNM staging system is used for risk stratification of the population, and cannot work as a tool to provide prognosis prediction for individual patients. Therefore, it cannot meet the need to improve patient prognosis. Today, with today’s increasingly perfect electronic medical record system, deep learning has been widely used in the medical field to predict the survival rate of cancer patients, which performs better than the traditional cox regression method (9–17). In this experiment, we trained a deep learning model based on a large amount of clinical data and developed a patient-oriented assistant utilizing this model. A recommendation system for radiotherapy and chemotherapy can be accessed through the Internet to provide patients with reference opinions for postoperative radiotherapy and chemotherapy regimens **Figure 1**.

## 2 Method

### 2.1 Eligibility criteria and patient information

Regarding the training cohort, We selected 4517 medical cases from Database: Incidence - SEER Research Plus Data, 18 Registries, Nov 2019 Sub (2000–2017) - Linked To County Attributes - Total U.S., 1969-2018 Counties, National Cancer Institute,DCCPS, Surveillance Research Program, released April 2021, based on the November 2019 submission. We included Data records if they meet the criterion (1), patients pathologically diagnosed between January 2012 and December 2017 with primary stage-teriii non-small cell lung cancer (NSCLC) and (2) the existence of one malignant lesion. On the contrary, We excluded clinical cases according to the standard (1), patients whose regional lymph nodes performed during the initial work-up or first course of therapy are unknown or missing. Then we choose the features relevant to the OS (overall survival) of the NSCLC, including demographic information (Age and Sex) and NSCLC-cancer-related characteristics (TNM stage, histology type, primary site, tumor size, regional node number examined, regional node positive number and laterality of the tumor), and treatment details(surgery of primary site, radiation, and chemotherapy), The outcome is the patient survival time and death indicator. As for the cohort for external validation of the model, the inclusion criteria and exclusion criteria are consistent with the training group, So we randomly collected 100 stage-iii non-small cell lung cancer patients who underwent surgery (Lobectomy WITH mediastinal lymph node dissection and Pneumonectomy) from January 2012 to December 2017 in Shaanxi Provincial People’s Hospital, China.

### 2.2 Data preprocessing and feature engineering

The training data and the testing data are stored in CSV files. Both datasets contain two types of variables in the covariates, numerical variable and categorical variable. In the dataset, we have 3 numerical variable fields, including regional node positive number, regional node number examined and tumor size as well as other 10 categorical variable features. In order to avoid the evaluation problems *via* using label encoding conversion to categorical, we converse the 10 categorical features by utilizing one hot encoding to identify the different categorical values in the feature in a binary fashion. To illustrate, Regarding feature surgery on the primary site, before conversion, this field contains two values encoded for two surgery types (Lobectomy WITH mediastinal lymph node dissection, Pneumonectomy WITH mediastinal lymph node dissection). After transformation, the very field will be replaced by two surgery types, the value of the two features could only be 0 or 1 to identify the specific surgery type. In addition, as for feature tumor size, in the training set the unit is millimeter while in the testing dataset, the unit is centimeter. So we divide the value in the training set by 10 to make the unit the same. Finally, we perform normalization in order to accelerate the training process.

### 2.3 Machine learning survival model design

In this section, we created three machine learning models to perform the survival analysis to select the optimal one.

We developed a deep learning model based on DeepSurv to predict personal hazard rate according to the patient’s current clinical condition. From the input to output, the patient’s baseline data is the input to the neural network, followed by the fully-connected hidden layers of nodes as well as a drop layer after each hidden layer. The output of the network is the hazard rate. Regarding the activation function of each node, in order to overcome the problem of vanishing gradients, we select ReLU to add nonlinearity to the model which could help the model learn the complex relationship between covariates and the hazard rate. As for the loss function, we train the model to minimize the average negative log partial likelihood with regularization:


(1) $$(\theta )=-\frac{1}{N_{E=1}}\sum_{i:Ei=1}\left({\hat{h}}_{\theta }(x_{i})-\log\sum_{j\in R(T_{i})}e^{{\hat{h}}_{\theta }(x_{j})}\right)+\lambda \cdot \left|\right|\theta {\left|\right|}_{2}^{2}$$

where *θ* is the weight of every node in the network, $\frac{1}{N_{E=1}}$ is the number of dead patients and *λ* is the *l*_2_ regularization parameter, ${\hat{h}}_{\theta }\left(x\right)$ is the predicated hazard rate. we use Adam for the gradient descent algorithm to update the parameter of the model for lots of epochs, because Adam is more efficient when working with problems involving high dimensional data and requiring less memory for optimization process compared with SGD method (18). We utilize random Search to optimize the hyper-parameters because compared to Grid Search, Random Search could try more cases for important hyper-parameters. In the experiment, we perform this on the log space of the learning rate in [0.00001, 0.1], the dropout rate in [0.2-0.5], the number of hidden layers in [1, 7] and the number of nodes in each hidden layer in [5,90].

We also trained a random forest model, this model is reliable because it forces each split to consider only a subset of the predictors. In this study, Random Search is still used to tune the number of the comprising trees in [100,300], the minimum number of samples required to split an internal node in [2,50] as well as the minimum number of samples required to be at a leaf node in [1,20].

Lastly, we trained the Penalized Cox Proportional hazard model with the same loss function as the deep learning model. we tuned the hyperparameter by using Random Search Method, specifically, the penalizer in [0.001,1] and the learning rate in [0.001,1].

### 2.4 Model training and evaluation

The concordance index(C-index) is used to measure the performance of the model. The C-index is the ratio of pairs of patients ordered correctly to all pairs. Thus the higher C-index, the better performance of the model. In the study, The 4517 SEER data records were divided into two groups, 3534(80%) records were used for training while 883(20%) records were treated as the validation set. The five-fold cross-validation was performed to tune the hyper-parameters of each model and select the best model for survival prediction. Additionally, external validation was performed on the selected optimal model and TNM staging system and compare the generalizability of the two models. Eventually, we performed the attribution analysis for the deep learning survival model by the integrated gradients (19) method based on the testing dataset to rank the clinical feature importance.

### 2.5 Cloud-based adjuvant therapy recommender system deployment

The deep learning algorithm could recommend treatment for patients according to their current clinical conditions (20). we could load the model and set the input according to the patient’s demographic feature(age and gender), Surgery Type(lobectomy and pneumonectomy), Type(histology type and laterality) and the stage information of NSCLC(TNM, the number of the examined regional node, the number of the positive regional node and the tumor size). As for Adjuvant therapy, we predict the hazard rate under four adjuvant therapy treatments (with radiation and chemotherapy, with radiation and without chemotherapy, without radiation and with chemotherapy, and without radiation and chemotherapy). Then we could get the four cumulative hazard functions under each adjuvant therapy treatment and finally derive the four 5-year survival functions after negating and exponentiating the cumulative hazard function. In this application, we develop the backend code to calculate the four 5-year adjuvant therapy survival functions and implement the UI code to display the predicated survival functions in the line race chart.

### 2.6 Computation software

The three models are trained with Python v 3.9, PyTorch v 1.11.0 is used to train the deep learning algorithm and PySurvival v 0.1.2 is utilized to train the random survival forest and penalized cox proportional hazard model. The Front UI of the adjuvant therapy recommender system is developed with Vue.js javascript framework and a Material Design component framework called Vuetify. The backend code of the web application is implemented by the Django REST framework. The recommender system is deployed on Tencent Cloud, which could be accessed through a web browser.

## 3 Results

### 3.1 Patient baseline characteristics

Based on the inclusion criteria, we include 4617 stage-iii NSCLC patients who received Surgeries (Lobectomy and Pneumonectomy with mediastinal lymph node dissection) in this study. The 4517 patients out of 4617 are extracted from the SEER database and used as a training set while the other 100 patients are from China Database for model testing. The baseline medical characteristics of the two cohorts are shown in **Table 1**. From the AJCC TNM staging system’s perspective, all patients in the training set and the testing set are stage-iii NSCLC patients. In the SEER cohort, most patients’ histology type is Adenocarcinoma, which takes 44.28%. The next one is Squamous cell carcinoma, which takes 23.27%. Regarding the Received surgeries, 85.51% of patients received Lobectomy WITH mediastinal lymph node dissection while the rest (14.48%) accepted Pneumonectomy WITH mediastinal lymph node dissection for treatment. Concerning Adjuvant treatment, 74.12% of patients accepted chemotherapy and about 41.88% received beam radiation. On the contrary, in the test cohort, most patients received Lobectomy WITH mediastinal lymph node dissection, the two leading histology types are Squamous cell carcinoma and Adenocarcinoma, respectively 46% and 43% of the population. As for Adjuvant treatment, 1/3 received beam radiation and almost everyone received chemotherapy.

**Table 1:** Main Baseline Clinical Characteristics of Patients.

Characteristic	Data set, No. (%)	
Age		
85+ years	60 (1.35)	0
80-84 years	242 (5.47)	0
75-79 years	530 (11.99)	2 (2.00)
70-74 years	731 (16.54)	10 (10.00)
65-69 years	891 (20.17)	14 (14.00)
60-64 year	713 (16.14)	22 (22.00)
55-59 years	573 (12.97)	22 (22.00)
50-54 years	395 (8.94)	20 (20.00)
45-49 years	171 (3.87)	6 (6.00)
40-44 years	63 (1.42)	3 (3.00)
35-39 years	25 (0.56)	1 (1.00)
30-34 years	13 (0.31)	0
25-29 years	7 (0.57)	0
20-24 years	0 (0)	0
15-19 years	3 (0.07)	0
Histologic type		
Neoplasm, malignant	6 (0.13)	0
Carcinoma, NOS	11 (0.24	0
Large cell carcinoma, NOS	45 (1.01)	1 (1.00)
Large cell neuroendocrine carcinoma	40 (0.90)	0
Large cell carcinoma with rhabdoid phenotype	1 (0.02)	0
Pleomorphic carcinoma	20 (0.45)	0
Giant cell carcinoma	6 (0.13)	0
Spindle cell carcinoma, NOS	4 (0.09)	0
Pseudosarcomatous carcinoma	13 (0.29)	0
Combined small cell carcinoma	16 (0.36)	0
Non-small cell carcinoma	114 (2.58)	0
Papillary carcinoma, NOS	3 (0.06)	0
Papillary squamous cell carcinoma	2 (0.04)	0
Squamous cell carcinoma, NOS	1028 (23.27)	46 (46.00)
Squamous cell carcinoma, keratinizing, NOS	76 (1.72)	1 (1.00)
Squamous cell carcinoma, large cell, nonkeratinizing, NOS	26 (0.58)	0
Squamous cell carcinoma, spindle cell	2 (0.04)	0
Lymphoepithelial carcinoma	4 (0.09)	0
Basaloid squamous cell carcinoma	7 (0.15)	0
Squamous cell carcinoma, clear cell type	3 (0.07)	0
Basaloid carcinoma	4 (0.09)	0
Adenocarcinoma, NOS	1956 (44.28)	43 (43.00)
Adenoid cystic carcinoma	6 (0.13)	0
Solid carcinoma, NOS	20 (0.45)	0
Carcinoid tumor, NOS	66 (1.49)	0
Neuroendocrine carcinoma, NOS	31 (0.70)	0
Atypical carcinoid tumor	31 (0.70)	0
Bronchiolo-alveolar adenocarcinoma, NOS	57 (1.29)	0
Alveolar adenocarcinoma	1 (0.02)	0
Bronchiolo-alveolar carcinoma, non-mucinous	4 (0.09)	0
Adenocarcinoma with mixed subtypes	277 (6.27)	1 (1.00)
Papillary adenocarcinoma, NOS	78 (1.76)	1 (1.00)
Clear cell adenocarcinoma, NOS	11 (0.24)	0
Mixed cell adenocarcinoma	11 (0.24)	0
Papillary microcarcinoma	1 (0.02)	0
Mucoepidermoid carcinoma	2 (0.04)	0
Mucinous adenocarcinoma	97 (2.19)	2 (2.00)
Mucin-producing adenocarcinoma	19 (0.43)	0
Signet ring cell carcinoma	5 (0.11)	0
Ductal carcinoma, micropapillary	2 (0.04)	0
Acinar cell carcinoma	162 (3.66)	0
Adenosquamous carcinoma	129 (2.92)	4 (4.00)
Adenocarcinoma with neuroendocrine differentiation	4 (0.09)	1 (1.00
Carcinosarcoma, NOS	4 (0.09)	0
Bronchiolo-alveolar carcinoma, mucinous	7 (0.16)	0
Bronchiolo-alveolar carcinoma, mixed mucinous and non-mucinous	4 (0.09)	0
T stage		
T1	0	2 (2.00)
T1NOS	2 (0.05)	0
T1a	371 (8.40)	1 (1.00)
T1b	390 (8.83)	0
T2NOS	25 (0.56)	0
T2a	1162 (26.31)	35 (35.00)
T2b	353 (7.99)	15 (15.00)
T3	1285 (29.09)	27 (27.00)
T3	828 (18.74)	20 (20.00)
TX	1 (0.02)	0
N stage		
N0	404 (9.14)	5 (5.00)
N1	866 (19.61)	11 (11.00)
N2	3087 (69.88)	84 (84.00)
N3	60 (1.36)	0
M stage		
M0	4417 (100.00)	100 (100.00)
Sex		
Female	2141 (48.47)	24 (24.00)
Male	2276 (51.52)	76 (76.00)
Radiation		
Beam radiation	1850 (41.88)	34 (34.00
Combination of beam with implants or isotopes	2 (0.05)	0
None	2412 (54.60)	66 (66.00
Radiation, NOS method or source not specified	14 (0.31)	0
Recommended, unknown if administered	88 (1.99)	0
Refused	49 (1.11)	0
Radioactive implants (includes brachytherapy)	2 (0.05)	0
Chemotherapy		
Yes	3274 (74.12)	95 (95.00)
No/Unknown	1143 (25.87)	5 (5.00)
Surgery to primary site		
Lobectomy WITH mediastinal lymph node dissection	3777 (85.51)	81 (81.00)
Pneumonectomy WITH mediastinal lymph node dissection	640 (14.48)	19 (19.00)
Laterality		
Left - origin of primary	1923 (43.53)	43 (43.00)
Only one side - side unspecified	1 (0.02)	0
Paired site, but no information concerning laterality	1 (0.02)	0
Paired site, but no information concerning laterality	2492 (56.41)	57 (57.00)

### 3.2 Training curve and model performance

**Figure 2:**
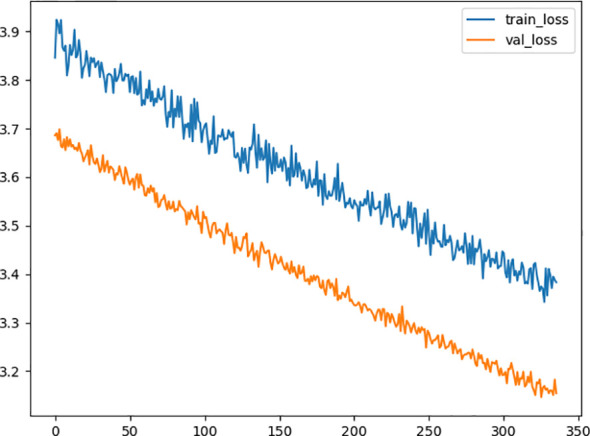
Diagram of the traing loss and the validation loss in the optimization procedure. The x-axis represents the number of epoch, and the y-axis represents value of loss function. The orange line is the validation loss function and the blue one represents the training loss function.

After the process of random search, we finally settled down on the hyperparameter of the deep learning model, the model consists of 2 hidden layers, from input to output, including 60, 43 neurons in each layer with a dropping out unit between each layer. we improve neural network generalization by setting the learning rate to 0.001 and 0.5 as the dropout rate to avoid overfitting. **Figure 2** shows the training loss curves of the survival network. At the beginning of the training process, the loss of the validation and training set decreases continually. After 331 epochs of parameter optimization, the loss of the validation set begins at 3.6936 and stops decreasing at 3.1753 while the training loss continues to decrease from 3.3844 started at 3.8446. Then we terminate the optimization to avoid overfitting and save the model for test.

In the random survival forest, We set the number of the estimating trees to 959, the minimum number of samples required to split an internal node to 10 and the minimum number of samples required to be at a leaf node to 15. In the Penalized Cox Proportional hazard model, we configure the penalizer to 0.005 and the learning rate to 0.01

**Figure 3:**
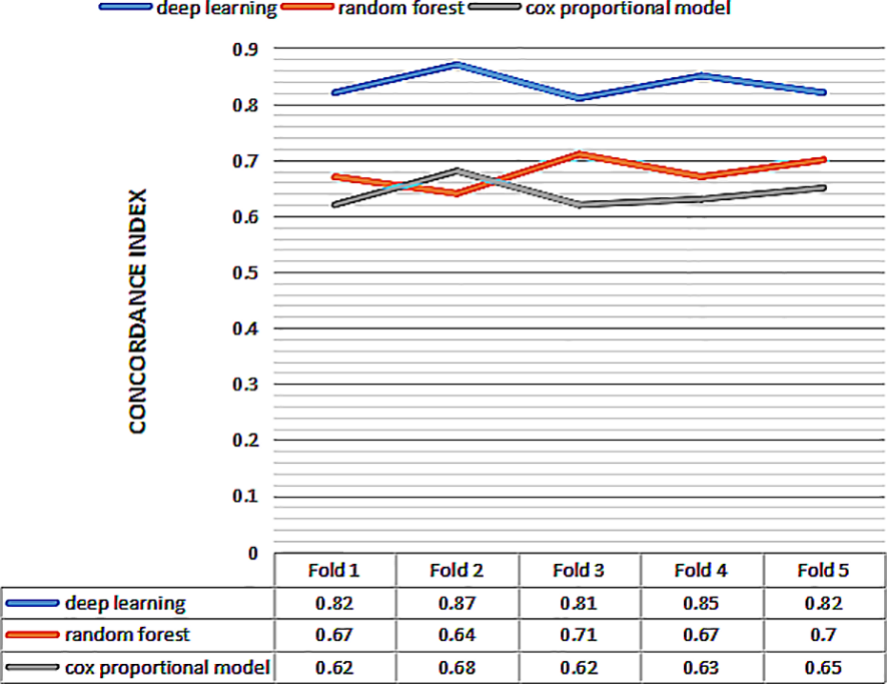
The concordance index of three models for 5 fold cross validation. The x-axis represents the number of fold, and the y-axis represents value of concordance index for each model.

Then we perform 5-fold cross-validation to select the optimal model for survival prediction. **Figure 3** displays the exact value and the line chart of each model in every fold validation, the deep learning model shows a more stable and exceptional performance on the concordance index compared to the other two models. The mean of the concordance index of the deep learning algorithm is 0.843, which is much higher than the random forest (0.678) and cox proportional hazard model (0.678) (**Table 2**). Based on the result of cross-validation, deep learning is selected to compare the TNM staging system on external validation. The performance of the deep learning model is better (0.82 vs 0.65)

**Table 2:** Performance of the survival models to predict hazard rate of the stage-III NSCLC patient received resection surgery.

MODEL	Cross Validation	External Validation
	Concordance Index Mean	Concordance Index
Deep Learning	0.834	0.820
Random Forest	0.678	
Cox Proportional	0.640	
TNM Staging		0.650

**Figure 4:**
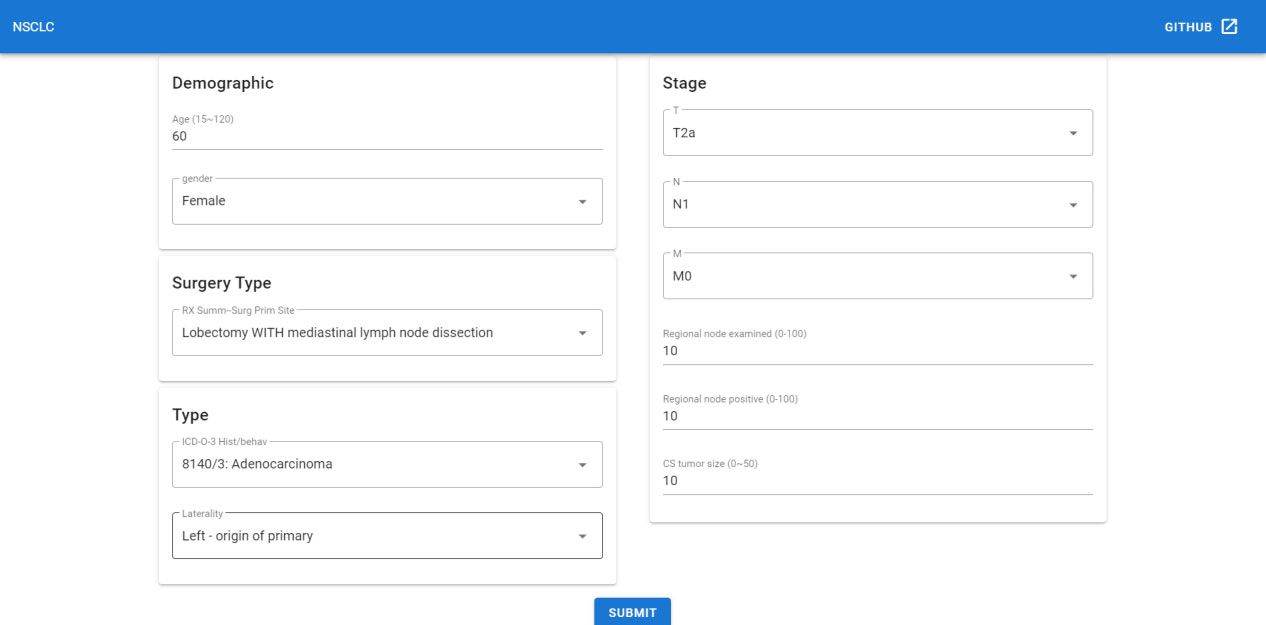
The attribution score of all input features in the deep learning model. The x-axis represents the name of the input features, and the y-axis represents value of attribution score for each feature.

As for the feature importance for the network, from the **Figure 4** we can observe four of the top important features: regional positive nodes (0.6634), regional examied nodes (-0.7648), tumor size (-0.5633) and Age(-0.4633). In terms of least important features, we observe that the surgery on the primary site (0.0632) is voted to be least significant based on attribution algorithm. The absolute value for attribution scores of other features is greater than 0.1 and less than 0.5.

Then we perform 5-fold cross-validation to select the optimal model for survival prediction. **Figure 3** displays the exact value and the line chart of each model in every fold validation, the deep learning model shows a more stable and exceptional performance on the concordance index compared to the other two models. The mean of the concordance index of the deep learning algorithm is 0.843, which is much higher than the random forest (0.678) and cox proportional hazard model (0.678) (**Table 2**). Based on the result of cross-validation, deep learning is selected to compare the TNM staging system on external validation. The performance of the deep learning model is better (0.82 vs 0.65)

### 3.3 The adjuvant therapy recommender system

**Figure 5:**
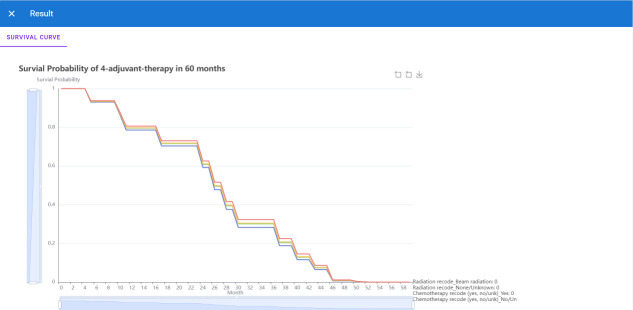
The input page of the recommender system.

Since the deep learning model has better performance than the TNM staging system, we could not only predict the survival function of the current patient but also offer an adjuvant therapy reference to the oncology doctor based on prediction over different therapy treatment plans. Thus we deployed the recommender system to the Internet, which could be accessed with a browser in [http://1.15.80.136/nsclc/], input the current clinical status, including Demographic, surgery type, cancer type and stage information, of one patient, and click the submit button (**Figure 5**).

**Figure 6:**
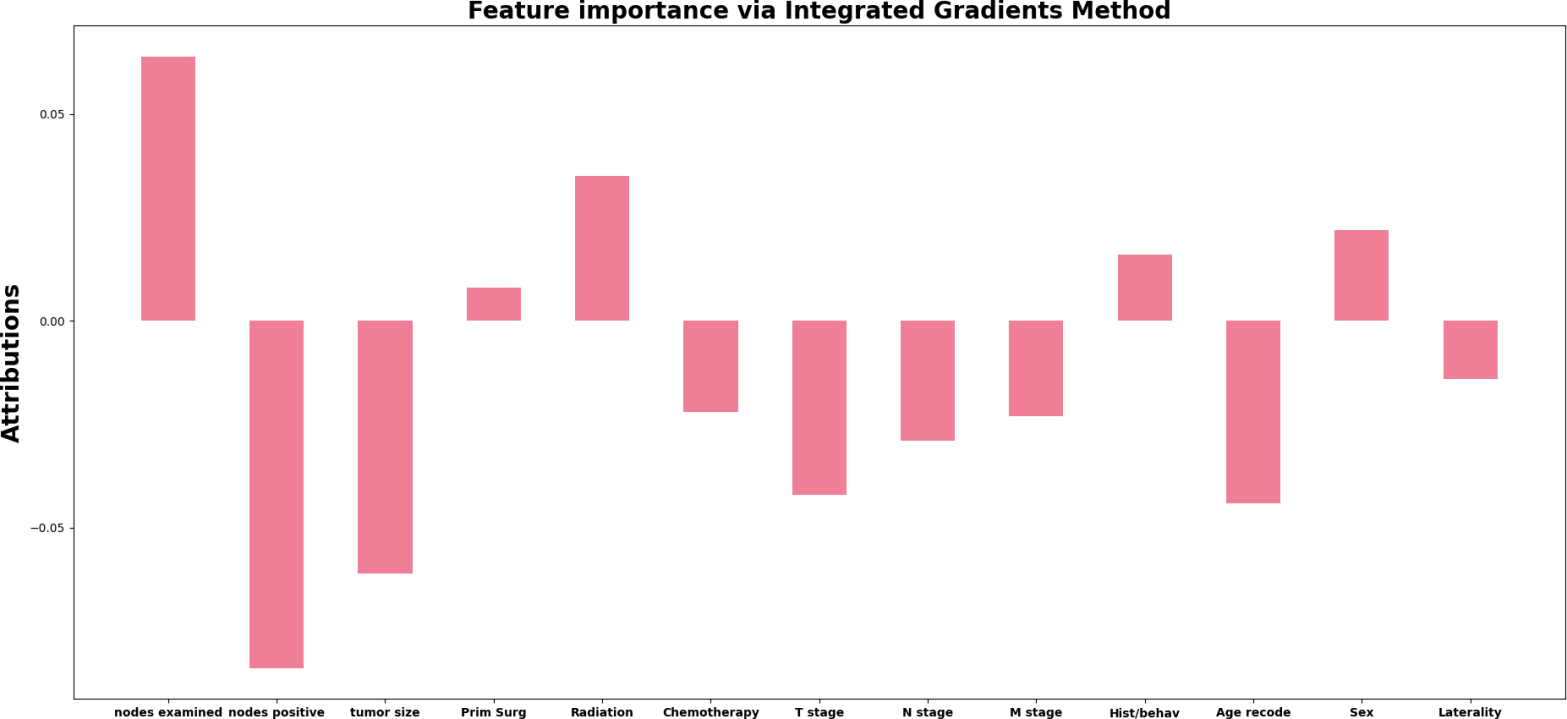
The output page of the recommender system.

Then the browser will redirect to the result page (**Figure 6**), and we could see four 5-year predicted survival curves for each treatment plan. Based on the plot, the predicted optimal treatment plan is only receiving beam radiation for adjuvant treatment, whose survival probability is highest in the next 60 months.

Thus, the specialist could get the reference for adjuvant treatment plan decision-making. Code related to this application can be found at https://github.com/snowflake-Zhao/nsclc.

## 4 Discussion

This study provides a model that is more accurate than the TNM staging system to predict the prognosis of the stage-iii received resection NSCLC cancer patients in 5 years. Additionally, the deep learning survival model is more precise and stable than the random survival forest and cox proportional model to predict the hazard rate of the stage-III resectable NSCLC cancer patients. This demonstrated our first goal that the deep learning approach is more reliable than TNM in predicting the hazard rate. Driven by the desire to resolve the controversy on devising adjuvant treatment plans for stage-iii received resection NSCLC cancer patients, we did solve this problem by developing a recommender system based on the externally validated deep learning model. To our best knowledge, this is the first recommender system to provide adjuvant treatment plans reference for stage-iii NSCLC cancer patients who received resection.

As reported, Adeoye J, et al. have trained DeepSurv and RSF (random survival forest) models for predicting the malignant transformation probability of oral leukoplakia and lichenoid lesions with (N=716) patients (21). Their exceptional results suggest a considerable improvement of accuracy for hazard prediction using the deep learning model when it is compared with the Cox proportional hazard model(C-index=0.95 vs 0.83), and RSF’s performance is much better and more stable than that of Cox proportional hazard model(C-index=0.91 vs 0.83) in this task. Our outcome of the experiment is consistent with their conclusion. In another study, Huang C, et al. developed software to select adjuvant radiotherapy and chemotherapy treatment plan according to the corresponding output hazard rate. Our software has two major points different from their product (22). One is the output page for oncology specialists. Their output is just one hazard rate, which is difficult for specialists and patients to understand. On the contrary, we plot the four adjuvant treatments predicted survival curves in 60 months, which is more straightforward for patients and doctors because people could understand their probability of survival for each adjuvant treatment plan in the 5 years. The other point is our software could be accessed directly through the web browser either on mobile phones, iPad or personal computers instead of installed on the personal computer for seeking recommendation guidance, which is not convenient for doctors to use.

In our study, the random survival forest did not perform well as Lin J, et al’s (C-index= 0.678 vs 0.723) (23), I think this is mainly because the two features in the dataset after one hot encoding, the Histologic type and Radiation, generate lots of sparse variables, including Radioactive implants, Signet ring cell carcinoma and so on, which eventually cause harm to the formation of different estimator trees. The result that the deep learning model’s C-index is higher than the Cox Proportional hazard model(C-index= 0.834 vs 0.640) meets our expectations, mainly because deep learning could formulate the complex relationships between clinical baseline characteristics and the patient’s hazard rate, which is more accurate than the linear relationship assumption of the Cox proportional hazard model. Additionally, the deep learning model has superior performance than the TNM staging system(C-index= 0.82 vs 0.65) is expected, because the neural network takes in more clinical features related to the prognosis of the patients, including Histologic type, age, sex, tumor size and many others, than the TNM staging system and the most important features of the network are regional postive nodes,regional examied nodes, tumor size and the Age, which is slightly different from the TNM stage system, even though the T stage value comes from the tumor size, N stage value comes form the regional nodes, we could tell the exact detailed number of the tumor size and the regional positive nodes could help the model to predict the prognoses more clearly than the general value. Besides the trained model could perform personal prognosis prediction while the TNM staging system could only predict the cohort prognosis. Thus, the deep learning model could possibly substitute the TNM staging system in the future if more medical records could be utilized for training.

In the current medical practice, there is a lack of consensus regarding the principles of adjuvant therapy for stage-iii NSCLC patients. For instance, According to the latest version of NCCN Guidelines for NSCLC(Version5.2022), one major controversy is inconsistent results among different randomized controlled trials of stage-III NSCLC (23–26). The one reason for the inconsistent results among different randomized controlled trials is the RCT lacks external validity (27), which means there might be neglected features that are effective for the prognosis. Because the externally validated deep learning model could include lots of features might related to the prognosis and be sensitive to the different inputs, the model could output the hazard risk of the different treatment plans, then the optimal plan could be obtained by comparing the output of different treatments. In our adjuvant recommendation system, we could obtain the reliable and accurate hazard rate for 4 adjuvant treatment plans from the developed externally validated model. To visualize the outcome, after mathematic transformation, the predicated survival curves for 4 treatment plans are displayed on the Web User Interface. Because of the significant prognostic benefit of following the treatment recommendation which clearly outweighs those who don’t, the recommendation system is promising to serve as a dependable tool for decision-making on the adjuvant treatment plan for each stage-iii NSCLC patient.

From the results of our experiment, the deep learning model performs well in the survival analysis task. However, the model is lacking in explainability owing to the high complexity inside the neural network, which is not realistic to explain the process to humans. If we want to extensively apply the deep learning algorithm in the decision-making of the NSCLC, we definitely need to improve the explainability of the model (28–30). we could incorporate the causal inference ideas in designing inherently interpretable models by adding sample reweighting technique into the loss function to compare the performance with our deep learning result in the future (31–34). Even though the SEER database has numerous NSCLC patient’s medical records, the database could record more detailed attributes in three aspects, including 1) resection information in detail, like resection status (R0/R1/R2) 2)detailed information related to beam radiation, for instance, total dose and dose per fraction 3) further information relevant to chemotherapy on drugs and dosage.

## 5 Conclusions

To our best knowledge, this study is the first to research the performance of a deep learning network and random forest in resected Stage-III NSCLC and obtain satisfactory results in survival prediction. In addition, the recommendation system for adjuvant therapy based on the deep learning model will be likely applied to offer recommendation reference to the specialist in the clinical practice.

## Data availability statement

The original contributions presented in the study are included in the article/**Supplementary Material**. Further inquiries can be directed to the corresponding author.

## Ethics statement

Written informed consent was obtained from the individual(s) for the publication of any potentially identifiable images or data included in this article.

## Author contributions

LJ and QZ designed the research. LJ collected the training and testing dataset. QZ trained the models and developed the web-application. LJ and QZ wrote the manuscript. JM, BH, SF and FC edited and critically revised the manuscript in regard to important intellectual content. All authors read and approved the manuscript.
